# Social media as a formative educational and cultural environment: an integrative conceptual framework for identity development among transnational youth

**DOI:** 10.3389/fsoc.2026.1834754

**Published:** 2026-07-21

**Authors:** Fouad Ahmed Atallah

**Affiliations:** Department of Sharia, College of Sharia and Law, Jouf University, Sakaka, Saudi Arabia

**Keywords:** algorithmic visibility, cultural negotiation, digital literacies, digital media, educational contexts, hybrid identities, identity development, multilingual practices

## Abstract

Social media has become a prominent context in which transnational youth negotiate identity, belonging, and meaning across cultural, linguistic, national, and educational boundaries. In this article, I examine how social media and digital media practices shape identity development among transnational youth, with particular attention to educational and cultural perspectives. Positioned as a conceptual research contribution, I ground this study in an analytical and interpretive synthesis of peer-reviewed scholarship drawn from four major databases (Google Scholar, Scopus, ERIC, and Web of Science), covering publications from 2006 to 2025. Following a structured three-stage thematic synthesis procedure, I retained 42 core studies from an initial pool of approximately 180 sources and analyzed them to develop an integrative conceptual framework. My analysis highlights how multilingual practices, translanguaging, counter-storytelling, hybrid identity construction, and strategic self-presentation across platforms enable youth to navigate multiple audiences and maintain simultaneous local, translocal, and transnational affiliations. Across the synthesized literature, I identify a consistent pattern indicating that social media functions as a “third space” that can bridge formal and informal learning contexts when educational institutions recognize the multilingual, multicultural, and digital literacies developed online. At the same time, I show that digital media practices are shaped by structural conditions—including unequal access to resources, platform governance, algorithmic visibility, surveillance, and geopolitical asymmetries—which may constrain identity development. I propose a triadic integrative model theorizing digital identity development as a recursive interaction between mediating mechanisms, structural conditions, and developmental outcomes. I conclude that social media should be understood not merely as a communicative tool but as a formative educational and cultural environment that plays a significant role in transnational youths’ identity development.

## 1 Introduction

In an era characterized by intensified global mobility, digital connectivity, and cultural interdependence, increasing numbers of young people grow up navigating lives that span multiple national, linguistic, and cultural contexts. These transnational youth experience identity development not within a single bounded social environment, but across overlapping social worlds shaped by migration, education, media, and everyday digital interaction (Lam, 2006; Wilding, 2012; Leurs, 2008). Within this landscape, social media has emerged as a central site through which transnational youth communicate, learn, and negotiate belonging, rendering digital media practices a significant force in contemporary identity development.

Research across sociology, education, linguistics, and media studies has increasingly recognized that identity is not a fixed attribute but a dynamic, socially mediated process shaped through interaction, discourse, and symbolic practice (Lam, 2014). Social media platforms extend these processes by enabling youth to maintain simultaneous affiliations with local, translocal, and transnational communities, often across considerable geographical and cultural distances. Through practices such as multilingual communication, code-switching, narrative self-presentation, and peer interaction, digital media allows young people to experiment with, contest, and reconfigure identities in ways that were not previously possible (Leurs and Ponzanesi, 2013; Yi, 2009).

A growing body of empirical research documents how digital media functions as a “third space” for transnational youth, offering opportunities for identity exploration beyond the constraints of home, school, and nation-state frameworks (Lam and Wu, 2024; Deroo and Mohamud, 2022). Studies have shown that platforms such as Facebook, Instagram, instant messaging applications, and culturally specific online spaces support hybrid identity formation by enabling youth to blend heritage and host cultural resources, sustain diasporic ties, and engage in counter-storytelling that challenges marginalizing representations (McKenzie, 2022; Mevsimler, 2021; Abokhodair, 2017). From an educational perspective, scholars have argued that these practices often constitute sophisticated forms of digital, linguistic, and cultural learning that remain underrecognized within formal schooling contexts (Darvin, 2018; Pang and Wang, 2020).

At the same time, research highlights significant tensions and debates within the field. While some studies emphasize the empowering potential of social media for voice, agency, and identity affirmation, others caution that digital environments can reproduce existing inequalities related to social class, migration status, language hierarchies, and access to resources (Leurs, 2008; Wilding, 2012). Divergent findings suggest that digital media does not uniformly enhance identity development; rather, outcomes are shaped by contextual factors such as generation of migration, cultural distance, institutional recognition, and platform-specific affordances (Kim, 2018; Yau et al., 2020). From a critical educational standpoint, scholars have further questioned uncritical celebrations of “digital literacy,” noting that policy and pedagogical discourses may obscure structural constraints and reinforce neoliberal assumptions about learning and connectivity (Darvin, 2018; Koyama, 2020).

Despite the richness of this literature, existing research remains fragmented across disciplines and often focuses on specific platforms, populations, or practices in isolation. There is a need for integrative analyses that bring together educational and cultural perspectives to clarify how social media shapes identity development among transnational youth more broadly. In particular, fewer studies synthesize empirical and theoretical insights to examine how digital media simultaneously enables learning, belonging, and hybrid identity formation while also constraining these processes through inequality and surveillance.

Responding to this gap, I examine the role of social media in identity development among transnational youth from educational and cultural perspectives. Drawing on an analytical and interpretive synthesis of interdisciplinary scholarship, I aim to identify key mechanisms through which digital media mediates identity formation and to clarify the conditions under which social media supports or constrains developmental outcomes. I argue that social media should be understood not merely as a communicative technology but as a formative educational and cultural environment. I conclude by highlighting implications for culturally responsive educational practices and for future research on youth, digital media, and identity in transnational contexts.

### 1.1 Positioning and contributions

Despite the growing body of research on social media and transnational youth, existing studies remain fragmented across disciplinary boundaries. Several important prior frameworks have addressed related dimensions of digital identity among transnational youth, yet each operates within a bounded disciplinary scope that generates specific analytical blind spots—blind spots that, taken together, point to the need for a framework that is not merely broader in coverage but differently structured in its explanatory logic.

Lam (2006, 2014) ecological framework for digital literacies and immigrant identity is foundational for understanding how youth develop new literacies in digitally mediated spaces. However, because it foregrounds practice and participation, it generates recommendations oriented primarily toward recognizing youth competencies within educational settings. It does not incorporate platform governance, algorithmic visibility, or structural constraints as variables that shape which practices are even possible—meaning a teacher or researcher using Lam’s framework alone would ask ‘what are students doing online?’ but would not ask ‘what is the platform allowing or preventing them from doing, and why?’ My model adds this second question as an analytically necessary complement to the first.

Darvin (2018) investment model advances a critical account of how social class and capital shape digital identity, producing important recommendations about reducing material inequalities in access. However, because the model is anchored in critical applied linguistics and focuses on capital as the key explanatory variable, it generates pedagogical recommendations that emphasize economic and social equity interventions. It does not integrate translanguaging practices, third-space dynamics, or transnationalism theory’s account of multi-sited identity. A practitioner using Darvin’s framework would focus on resource provision—but would not have a theoretical basis for recognizing how a student’s simultaneous multilingual affiliations across platforms constitute a distinct form of identity work that cannot be reduced to capital acquisition alone.

Leurs (2008) digital diasporas framework offers an indispensable sociological account of how diasporic youth negotiate identity across digital platforms, with particular attention to generational and gender dynamics. However, because it foregrounds sociological rather than educational dimensions, it does not integrate formal learning literatures, translanguaging research, or pedagogical implications. A researcher using Leurs’ framework would produce rich accounts of youth digital culture—but would not generate the bridge to classroom practice or curriculum design that an education-facing framework requires.

Lam and Wu (2024) chronotopic framework is the most recent and theoretically sophisticated of the four, offering important advances in literacy theory through its attention to the spatiotemporal dimensions of digital practice. However, it does not incorporate the structural conditions—platform governance, algorithmic visibility, surveillance, geopolitical asymmetries—that shape which chronotopes are accessible to which youth under which constraints. A researcher using this framework would generate rich interpretive accounts of how youth imagine and inhabit social worlds—but would not produce the structural critique necessary for policy-oriented conclusions.

What my model adds is not a set of new empirical variables, but a different explanatory architecture. By constructing a triadic framework in which mediating mechanisms, structural conditions, and developmental outcomes are theorized as recursively interdependent, the model generates analytical moves that none of the prior frameworks support on their own. Specifically, my framework produces three kinds of explanation that the prior frameworks do not individually yield:

- It explains why the same digital practice—for example, translanguaging on TikTok—can produce different developmental outcomes for different youth, by theorizing structural conditions (algorithmic visibility, surveillance, platform governance) as a variable layer that mediates between practice and outcome. Lam’s framework explains what the practice is; Darvin’s explains how capital shapes access to it; my model explains why the same practice yields empowerment for some youth and marginalization for others within the same platform environment.
- It generates pedagogical recommendations that are simultaneously practice-oriented and structurally critical—something neither Lam nor Leurs nor Darvin alone supports. A teacher working with my framework would not only be asked to recognize students’ multilingual digital competencies (Lam), or to address resource inequalities (Darvin), or to understand diasporic dynamics (Leurs), but also to teach students to critically examine platform governance and algorithmic visibility as structural determinants of whose voices are heard—a recommendation that emerges specifically from the three-way interaction among the model’s components.
- It accounts for the recursive dimension of identity development over time—where outcomes reshape subsequent practices, which encounter changed structural conditions—in a way that linear acculturation or capital-acquisition models cannot. This recursive logic is particularly important for longitudinal research designs and for understanding why identity trajectories diverge in ways that static models cannot predict.

I acknowledge that this model is primarily integrative in character rather than structurally unprecedented. The individual components—practice, structure, outcome—are recognizable across the literature. The claim to originality rests on three grounds: the specific architecture of their recursive interdependence; the explicit application of that architecture to both educational and migration-focused contexts simultaneously; and the consequential difference in explanation and pedagogy that this architecture produces relative to each prior framework taken individually. I defend this integrative contribution as substantive precisely because the fragmentation it addresses is not merely a gap in coverage but a gap in explanatory capacity—one that has produced frameworks well suited to their home disciplines but insufficient for the transdisciplinary, structurally attentive, and educationally applicable account that the field requires.

## 2 Literature review

### 2.1 Theoretical foundations

The conceptual framework I develop in this article rests on four complementary theoretical traditions that together provide a robust and explicitly stated foundation for understanding identity as a dynamic, socially mediated, and power-inflected process in digital environments.

*Sociocultural Theory*: Drawing on the foundational work of Vygotsky (1978) and Wertsch (1991), sociocultural theory conceptualizes identity as mediated by cultural tools, social interaction, and participation in communities of practice. Within this framework, I theorize digital media platforms as contemporary mediational tools through which identity-related learning and negotiation occur. Translanguaging, counter-storytelling, and strategic self-presentation are understood as tool-mediated practices through which transnational youth act on and within their social worlds. This perspective is central to my understanding of digital media as a formative educational environment rather than a passive channel of communication.

*Social Identity Theory*: Drawing on Tajfel and Turner (1979, 1986), I incorporate the insight that identity is constituted through social categorization, group membership, and intergroup comparison. For transnational youth, social media extends the arenas in which group membership and identity boundaries are negotiated, contested, and performed. The theory helps explain why platform choices, language selections, and self-presentation strategies are identity-consequential acts rather than merely communicative ones, and why belonging and recognition—or their denial—carry significant psychological and developmental weight.

*Poststructuralist Approaches to Identity*: Drawing on Hall (1990), Butler (1990), and Norton (2000), poststructuralist perspectives understand identity as non-unitary, discursively constituted, and enacted through performative practices. This perspective grounds my treatment of identity as fluid, situationally activated, and subject to ongoing renegotiation through digital discourse. It also provides the theoretical basis for understanding counter-storytelling and strategic self-presentation not as distortions of a stable self, but as legitimate and constitutive performances of identity in contexts shaped by unequal power relations (Cordova et al., 2010).

*Transnationalism Theory*: Drawing on Vertovec (2009) and Glick Schiller et al. (1992), transnationalism theory conceptualizes transnational youth as social actors whose lives, practices, and identities span multiple nation-states simultaneously, operating within social fields that cross geopolitical borders. I position digital media, within this framework, as a key infrastructure through which transnational social fields are maintained, navigated, and reproduced. This perspective is essential for understanding why digital identity work among transnational youth is not simply about individual preference or cultural mixing, but about navigating structurally positioned social worlds with competing demands, loyalties, and possibilities (Kim, 2015; Eide et al., 2014).

Together, these four theoretical traditions provide the conceptual scaffolding for the integrative model I propose in Section 4.7. I weave them into the analysis throughout Sections 4 and 5 to ensure that theoretical engagement is not confined to a prefatory section but informs the interpretation of specific patterns and mechanisms identified in the synthesis.

### 2.2 Digital media and identity development among transnational youth

A substantial body of empirical and theoretical research has examined how social media and digital media practices shape identity development among transnational youth across diverse cultural and educational contexts. Grounded in the sociocultural and poststructuralist traditions outlined above, these studies consistently conceptualize identity as a dynamic and socially mediated process that unfolds through interaction, discourse, and participation in networked environments (Lam, 2006; Yi, 2009; Leurs, 2008). For transnational youth—whose lives are shaped by migration, mobility, and multilingualism—digital media has become a particularly salient arena for negotiating cultural, ethnic, national, religious, and gender identities.

Empirical studies employing qualitative, ethnographic, and mixed-methods approaches demonstrate that social media enables transnational youth to sustain simultaneous affiliations with local immigrant communities, translocal ethnic networks, and transnational peer groups in countries of origin (Wilding, 2012; Lam, 2009; Kim, 2018). Platforms such as Facebook, Instagram, and instant messaging applications facilitate ongoing cross-border communication and identity experimentation, allowing youth to navigate multiple social worlds that extend beyond the spatial and institutional boundaries of home and school (Yau et al., 2020; Pang and Wang, 2020).

### 2.3 Digital media as “third spaces” for identity work

One of the most influential conceptual contributions in this field is the framing of digital media as “third spaces” for identity development. Drawing on sociocultural and literacy theories, scholars argue that online environments function as spaces where transnational youth can hybridize identity resources drawn from heritage and host cultures, creating new forms of belonging that resist rigid national or cultural categorizations (Lam, 2006; Leurs and Ponzanesi, 2013; Lam and Wu, 2024). These digital third spaces enable youth to engage in dialogic identity construction through interaction with diverse audiences, peers, and cultural texts.

Research with refugee youth, immigrant adolescents, and international students illustrates how digital media supports identity negotiation beyond institutional constraints by allowing youth to experiment with self-representation, language choice, and narrative positioning (Wilding, 2012; Koyama, 2020; McKenzie, 2022). The concept of transworlding further captures how youth navigate multiple imagined and lived social worlds through digital practices, simultaneously engaging with content from home countries while participating in educational and social contexts in host societies (Koyama, 2020).

### 2.4 Linguistic practices, counter-storytelling, and identity management

Across the literature, multilingual practices emerge as a central mechanism through which digital media mediates identity development. Studies document how transnational youth employ code-switching, translanguaging, and hybrid language forms to negotiate audience expectations and express multifaceted identities online (Lam, 2009; Darvin, 2018; Abdi, 2016). These practices are not merely communicative strategies but are deeply tied to processes of belonging, legitimacy, and identity affirmation—dimensions that are theorized within the poststructuralist tradition as performative acts of identity constitution (Ibold, 2010).

Another prominent theme concerns counter-storytelling as a form of resistance to marginalizing representations. Research on Muslim youth, African immigrant youth, and British Somali women shows how social media enables users to challenge dominant media narratives and articulate alternative identity positions through storytelling, activism, and discursive reframing (Deroo and Mohamud, 2022; Mevsimler, 2021; Nantwi et al., 2017). In addition, studies of international students identify strategic identity management practices—such as boundary management and selective self-presentation—that help youth navigate context collapse and multiple audiences across platforms (Yau et al., 2020; Xu, 2022; de Kool, 2012).

### 2.5 Cultural negotiation, generational differences, and inequality

While many studies highlight the empowering potential of digital media, the literature also reveals significant tensions and divergent findings. Identity outcomes vary depending on contextual factors such as migration generation, access to resources, cultural distance, and societal reception of migrant groups. Second-generation youth often demonstrate more hybrid and flexible identity formations, whereas first-generation migrants report greater identity tensions and feelings of dissonance (Kim, 2018; Leurs, 2008)—a pattern consistent with social identity theory’s account of intergroup positioning and perceived threat. Generational differences within families further shape digital practices, with youth frequently acting as cultural and technological brokers between heritage and host contexts (Leurs and Ponzanesi, 2013).

Critical perspectives emphasize that digital media can reproduce existing social inequalities. Studies drawing on Bourdieu’s theory of social and cultural capital demonstrate that youth with greater access to digital resources and diverse social networks benefit more from online identity work, while marginalized youth face constraints related to surveillance, platform hierarchies, and linguistic or racialized discrimination (Darvin, 2018; Wilding, 2012). Recent scholarship has extended postcolonial frameworks to examine how global digital platforms may contribute to cultural dependency and new forms of digital colonization, particularly among youth from the Global South (Sarnou, 2025).

### 2.6 Educational implications and ongoing debates

From an educational perspective, research indicates that digital media practices can bridge formal and informal learning contexts when institutions recognize the multilingual, multicultural, and digital literacies that transnational youth develop online (Lam, 2014; Eamer et al., 2014). Studies in K–12 and higher education settings show that social media can support intercultural competence, critical dialogue, and identity exploration when integrated intentionally into pedagogical practices (Pang and Wang, 2020; McKenzie, 2022).

However, scholars caution against uncritical celebrations of digital literacy. Educational policies and pedagogical discourses that ignore structural inequalities may inadvertently reinforce neoliberal assumptions about learning and connectivity, overlooking the diverse material conditions shaping youth digital engagement (Darvin, 2018; Koyama, 2020). As a result, the literature calls for more integrative and critically informed analyses that connect digital media practices, identity development, and education within broader cultural and institutional frameworks.

### 2.7 Synthesis and research gap

Overall, the existing literature indicates that social media plays a significant but complex role in shaping identity development among transnational youth, and that existing frameworks approach this complexity from within bounded disciplinary perspectives. As I document in Section 1.1, no prior model integrates mediating mechanisms, structural conditions, and developmental outcomes across both educational and migration-focused traditions. I respond to this gap through an analytical synthesis that bridges these strands of research, foregrounding implications for culturally responsive education in digitally mediated, transnational contexts. The following sections document my methodological approach and present the integrative thematic analysis.

## 3 Materials and methods

### 3.1 Study design and article classification

This article is a conceptual research contribution in which I develop an original theoretical argument through a structured interpretive synthesis of interdisciplinary scholarship. This positioning distinguishes my approach from a descriptive literature review, a systematic review, or a meta-synthesis: I treat the reviewed literature not as an object of descriptive enumeration, but as analytical material used to construct an integrative conceptual framework explaining how digital media mediates identity development among transnational youth within educational and cultural contexts.

Methodologically, I adopt a qualitative theory-building design based on analytical literature synthesis (Jabareen, 2009; Torraco, 2005). This approach is appropriate for fields characterized by conceptual fragmentation and interdisciplinary dispersion, where theoretical integration can provide significant scholarly value independent of primary data collection. Rather than aiming for exhaustive coverage or statistical aggregation, I focus the research design on identifying recurring mechanisms, structural conditions, and developmental outcomes that emerge across diverse empirical and theoretical studies.

### 3.2 Literature search strategy and corpus construction

I developed the analytical corpus through a purposive and iterative literature search focusing on peer-reviewed scholarship in education, sociology, media studies, sociolinguistics, and youth studies. I conducted searches across four major academic databases: Google Scholar, Scopus, ERIC (Education Resources Information Center), and Web of Science. I covered publications from 2006 to 2025, with foundational works published prior to 2006 included where they provided essential theoretical grounding (e.g., Lam, 2006; Leurs, 2008; Vygotsky, 1978; Tajfel and Turner, 1979).

I focused the search strategy on studies addressing the intersection of digital or social media, identity development, and transnational youth within educational or cultural contexts. Table S1 (Supplementary Materials) documents the complete search strategy, including databases, Boolean search strings, and term combinations. I used the following key search terms in combination: social media; digital media; identity development; transnational youth; migrant youth; immigrant youth; refugee youth; international students; digital identity; multilingual practices; translanguaging; platform governance; algorithmic visibility; education; learning; cultural identity. I combined keyword searching with backward reference tracking of influential studies and recent publications.

The initial database search yielded approximately 180 potentially relevant sources. Following title and abstract screening against the inclusion criteria described in Section 3.3, I retained 78 sources for full-text review. After full-text assessment for analytical relevance, depth, and scholarly quality, I retained 42 core studies as the primary analytical corpus. I included foundational works frequently cited in the field alongside more recent contributions to ensure both theoretical grounding and contemporary relevance.

I continued the iterative process until I reached conceptual saturation. I operationalized conceptual saturation as the point at which additional sources reviewed did not introduce new analytical categories or substantially alter the emerging thematic structure—a criterion grounded in established qualitative theory-building methodology (Jabareen, 2009; Torraco, 2005). I acknowledge that conceptual saturation in synthesis-based research is inherently judgment-based and subject to the limitations of corpus scope, which I note as a limitation in Section 5.6.

### 3.3 Inclusion and exclusion criteria

I included studies if they met the following conditions:

- Peer-reviewed empirical or theoretical publications;
- Focus on transnational, migrant, immigrant, refugee, or internationally mobile youth populations;
- Explicit engagement with social or digital media practices;
- Direct analytical focus on identity development, identity negotiation, or identity construction (including cultural, ethnic, national, linguistic, religious, or hybrid identities);
- Clear relevance to educational, learning, or cultural processes.

I excluded studies if they:

- Addressed technology use without substantive identity analysis;
- Focused exclusively on non-transnational populations;
- Lacked analytical depth or scholarly rigor;
- Consisted of non-scholarly or purely descriptive reports.

Following screening and full-text assessment, I retained 42 analytically relevant studies. Table A2 (Appendix B) provides a complete summary of all 42 studies in the analytical corpus.

### 3.4 Analytical procedure

I examined the selected studies using a thematic synthesis approach oriented toward conceptual integration and theory development (Thomas and Harden, 2008). The analytical process followed three stages, the outputs of which correspond directly to the structure of Section 4.

*Stage 1—Deductive coding*: I conducted an initial deductive coding phase using sensitizing concepts drawn from the four theoretical traditions outlined in Section 2.0—digital third spaces, identity negotiation, hybrid identity formation, transnational literacies, and digital belonging. I applied these concepts as initial analytical lenses systematically to each source in the corpus.

*Stage 2—Inductive pattern analysis*: I conducted inductive analysis to identify recurring patterns across studies related to mediating practices, structural conditions, identity processes, and educational implications. This phase involved close reading of each source, systematic annotation of key arguments and empirical findings, and analytical memo-writing to capture emerging conceptual connections across studies. I assigned codes independently to each source and compared them iteratively across the corpus.

*Stage 3—Constant comparative refinement*: I refined themes through constant comparison, identifying convergences, tensions, and gaps across sources, and ensuring that emerging categories were grounded in the breadth of the corpus. This stage produced five thematic clusters that form the structure of Section 4: (i) identity dimensions negotiated through digital media; (ii) digital media as third spaces for identity construction; (iii) linguistic, narrative, and self-presentation practices; (iv) enabling and constraining structural conditions; (v) educational implications for learning and pedagogy. I then synthesized these five clusters into the integrative triadic model in Section 4.7, which represents the conceptual culmination of the three-stage analytical process.

### 3.5 Analytical rigor and transparency

I used several strategies to enhance analytical rigor: explicit inclusion criteria; iterative cross-corpus comparison; identification of patterns recurring across diverse contexts; documentation of the corpus in Appendix B; and explicit justification of conceptual saturation. I do not aim for exhaustive coverage, but rather for theoretically coherent synthesis aligned with my conceptual objectives (Jabareen, 2009; Dekker, 2018).

### 3.6 Positionality statement

As a scholar working at the intersection of education, digital media, and sociocultural theory, I bring a particular disciplinary standpoint to this synthesis. My primary training is in education and educational sociology, which shapes the analytical weight I assign to formal learning contexts, pedagogy, and institutional recognition of youth competencies. I have engaged extensively with transnational and migrant youth populations through educational research, which has sensitized me to the structural inequalities shaping their digital and learning experiences. At the same time, I acknowledge that my positioning as a researcher based in an institution in the Arab world—where youth digital practices, platform governance, and geopolitical asymmetries take on specific regional dimensions—may have both enriched and bounded my analytical gaze.

In constructing the analytical corpus, I prioritized studies with explicit theoretical and educational dimensions, which may have led me to underweight scholarship foregrounding purely sociological, psychological, or computational perspectives. I have sought to counterbalance this tendency by deliberately including studies from disciplines beyond education—media studies, sociolinguistics, migration sociology, and critical digital media studies—and by centering structural and critical perspectives in Sections 4.5 and 5.4. I present the integrative model proposed in Section 4.7 as a theoretically grounded synthesis shaped by these disciplinary commitments, and I invite future researchers to test, extend, and critique it from standpoints and contexts that differ from my own.

## 4 Results

In this section, I present the five thematic clusters derived from the analytical synthesis, organized to capture the mechanisms, conditions, and processes through which social media shapes identity development among transnational youth. Each subsection draws on the reviewed literature as analytical material, and concludes with a brief transitional statement connecting the theme to the subsequent analysis. Across the synthesized scholarship, I consistently find that digital media tends to function as a complex formative environment that both enables and constrains identity development, with outcomes varying according to contextual, cultural, and educational factors (Lam, 2006; Leurs, 2008; Wilding, 2012; Yau et al., 2020).

### 4.1 Identity dimensions negotiated through digital media

Across the reviewed studies, I identify a consistent pattern in which transnational youth negotiate multiple and overlapping dimensions of identity through social media and digital platforms. Reflecting the poststructuralist and transnationalism theoretical traditions outlined in Section 2.0, I understand these identity dimensions not as fixed or essentialized categories, but as fluid, relational, and situationally activated positions shaped through interaction, discourse, and platform-specific practices (Lam, 2009; Kim, 2018; Boumba, 2014).

The most frequently documented identity dimensions include: cultural and ethnic identity, involving exploration, affirmation, and hybridization of heritage and host cultural resources; national identity, expressed through alignment, distancing, or rearticulation of belonging across nation-states; religious identity, particularly salient among Muslim youth engaging in counter-narratives and ethical self-representation; gender identity, negotiated through selective visibility and resistance to normative expectations; and hybrid or multiple identities, reflecting flexible positioning across audiences and communicative contexts (Abokhodair, 2017; Leurs and Ponzanesi, 2013; Mevsimler, 2021). The synthesized scholarship suggests that social media enables youth to sustain simultaneous affiliations with local, translocal, and transnational communities, with identity positions shifting depending on platform affordances, audience composition, and communicative purpose (Lam and Wu, 2024; Nantwi et al., 2017; Zhao, 2023).

The multiplicity of identity dimensions negotiated online points toward the central role of digital platforms as identity-constitutive spaces—a theme I develop further in the analysis of third spaces that follows.

### 4.2 Digital media as “third spaces” for identity work

A central pattern I identify across the synthesized literature is the conceptualization of digital media as “third spaces” extending beyond the institutional boundaries of home, school, and nation-state frameworks. Grounded in the sociocultural tradition’s emphasis on mediated participation in communities of practice (Vygotsky, 1978; Wertsch, 1991), these spaces appear to enable transnational youth to draw on diverse linguistic, cultural, and symbolic resources to experiment with self-representation, belonging, and social positioning (Lam, 2006; Koyama, 2020; Mallos, 2020; Prinsen et al., 2015).

Digital platforms function as dialogic environments in which identity is co-constructed through interaction with peers, family members, and imagined audiences across geographical distances. These third spaces appear to enable the blending of heritage and host cultural practices, participation in transnational peer networks, engagement with cultural texts from multiple sociocultural contexts, and reflexive positioning in relation to dominant and marginalizing discourses (Leurs, 2008; Lam and Wu, 2024; McKenzie, 2022). The synthesized scholarship indicates that such third spaces are particularly significant for youth navigating restrictive or exclusionary offline environments, offering alternative sites for voice, agency, and identity exploration (Wilding, 2012; Deroo and McClure, 2024; Solmaz, 2020; Hinton and Putra, 2020).

While third spaces offer significant identity-developmental opportunities, the linguistic practices through which youth navigate them are themselves analytically important—as the following section demonstrates.

### 4.3 Linguistic practices and identity mediation

Multilingual practices emerge across the corpus as a core mechanism through which digital media mediates identity development. From a sociocultural perspective, I interpret these practices as the deployment of linguistic tools in the service of identity-related social action (Vygotsky, 1978). Across contexts, transnational youth engage in code-switching, translanguaging, and hybrid language forms to navigate audience expectations and to index multiple identity positions simultaneously (Abdi, 2016; Lam, 2014).

These linguistic practices serve several identity-related functions: affirming affiliation with heritage and diasporic communities; signaling competence and legitimacy within host-society peer groups; managing boundaries between family, school, and peer audiences; and enabling flexible identity positioning across platforms characterized by different norms and visibility regimes (Lam, 2009; Kim, 2018). The synthesized literature indicates that language choice in digital environments is rarely neutral; instead, it functions as a semiotic resource through which youth negotiate belonging, authority, and social recognition (Darvin, 2018; Xu, 2022; Yoon, 2016).

These linguistic practices do not unfold in a vacuum: they intersect with discursive strategies of resistance and self-presentation that constitute a further dimension of digital identity work.

### 4.4 Counter-storytelling and strategic identity management

Another prominent pattern I identify across the corpus concerns the role of counter-storytelling in digital identity work. Grounded in poststructuralist understandings of identity as performatively constituted through discourse (Hall, 1990; Butler, 1990), counter-storytelling represents an active deployment of narrative to contest dominant representations and articulate alternative identity positions. Marginalized transnational youth—particularly Muslim youth and racialized immigrant groups—use social media to challenge negative representations circulating in mainstream media and public discourse (Abokhodair, 2017; Sarnou, 2025; Willem, 2010).

Counter-storytelling practices include producing alternative narratives about religion, culture, and migration; engaging in digital activism and justice-oriented literacies; and reframing stigmatized identities through humor, narrative, and visual representation (Nantwi et al., 2017; McKenzie, 2022). In parallel, I document sophisticated forms of strategic identity management in the synthesized studies, whereby youth curate self-presentation across platforms to manage context collapse and multiple audiences. These strategies include boundary management, selective visibility, and differentiated platform use depending on perceived risks and platform affordances (Yau et al., 2020; Pang and Wang, 2020; McDaniel, 2024).

The capacity of transnational youth to engage in these practices, however, is fundamentally shaped by the structural conditions examined in the following section.

### 4.5 Enabling and constraining conditions of identity development

My synthesis reveals that identity-related outcomes of digital media use are highly contingent on structural conditions. While many studies highlight empowering possibilities, others document significant constraints (Leurs, 2008; Darvin, 2018). A balanced analytical account requires equal attention to both—a requirement grounded in the critical-sociological dimensions of the transnationalism and poststructuralist traditions.

#### 4.5.1 Enabling conditions

I identify key enabling conditions as: access to digital resources and stable connectivity, diverse and supportive social networks, educational recognition of multilingual and digital literacies, and opportunities for reflexive and critical media engagement (Lam, 2014; Koyama, 2020). Youth with greater digital capital—encompassing devices, connectivity, and the social and cultural competencies needed to leverage digital environments effectively—are better positioned to convert digital participation into meaningful identity exploration and learning (Baan, 2020).

#### 4.5.2 Platform governance and algorithmic visibility

Commercial social media platforms are governed by content moderation policies, recommendation algorithms, and engagement logics that systematically advantage certain voices, languages, and identity performances over others (Leurs, 2008; Gillespie, 2018). For transnational youth, algorithmic visibility operates as an invisible form of hierarchization, amplifying content that conforms to dominant cultural norms while marginalizing multilingual, non-Western, or minority-language expression. Automated content moderation systems encode cultural biases that reflect the values and norms of their predominantly Western designers, resulting in differential moderation outcomes for racialized and linguistically minoritized youth (Noble, 2018; Eubanks, 2018).

Research has shown that racialized and linguistically minoritized youth face disproportionate risks of content removal, shadowbanning, and algorithmic invisibility on major platforms, effectively constraining the counter-storytelling and identity affirmation practices I document in Section 4.4 (Sarnou, 2025; Darvin, 2018). These dynamics are rarely transparent to users and are shaped by geopolitical and commercial interests that extend well beyond the individual user’s agency.

#### 4.5.3 Surveillance, datafication, and self-censorship

Surveillance by multiple actors constitutes a further structural constraint. I document three overlapping forms in the corpus: familial surveillance, in which parents or extended family members monitor youth digital activity; institutional surveillance, in which schools, universities, or immigration authorities monitor social media with potential consequences for students’ academic standing or legal status; and platform-level datafication, in which commercial platforms collect and monetize behavioral data in ways that shape what content is produced, retained, and made visible.

The anticipation of surveillance—whether or not it materializes—produces documented effects of self-censorship, code-switching, and strategic concealment among transnational youth (Abokhodair, 2017; Wilding, 2012). Youth from politically sensitive migration backgrounds, those with insecure legal status, and those from communities subject to security-oriented monitoring report greater degrees of self-censorship, correspondingly limiting their capacity for open identity exploration.

#### 4.5.4 Geopolitical asymmetries and digital colonization

Dominant social media platforms—predominantly developed and governed in the United States—encode cultural assumptions, interface designs, and moderation norms that may not reflect the communicative repertoires or political realities of youth in the Global South (Sarnou, 2025). Postcolonial and critical digital media scholars argue that such architectures contribute to new forms of “digital colonization,” in which youth from the Global South are positioned as consumers and data sources within infrastructures designed primarily for Northern users (Sarnou, 2025; Leurs, 2008). This dynamic reinforces existing hierarchies of cultural worth and linguistic prestige, shaping which identity performances are legible, valued, and rewarded on global platforms.

#### 4.5.5 Generational and intersectional variation

Constraining conditions interact with individual and group-level factors. Generational differences shape outcomes, with second-generation youth more frequently exhibiting hybrid identity formations and first-generation migrants reporting greater constraints (Leurs and Ponzanesi, 2013; Kim, 2018)—a pattern consistent with social identity theory’s account of shifting intergroup positions across generational contexts (Tajfel and Turner, 1986). Intersectional factors—including gender, religion, socioeconomic class, and migration status—mediate how conditions are experienced, such that no single structural account adequately captures the diversity of digital identity experiences.

The cumulative effect of these enabling and constraining conditions on educational participation and learning is addressed in the following section.

### 4.6 Educational implications identified in the literature

The synthesized studies indicate that social media can bridge formal and informal learning contexts when institutions recognize the competencies youth develop online, including language development, intercultural competence, and critical literacy (Eamer et al., 2014; Nantwi et al., 2017). However, I also consistently identify a disconnect between youth digital practices and dominant educational frameworks in the synthesized scholarship—a gap that uncritical conceptions of digital literacy risk widening by obscuring structural inequalities and undervaluing the complex identity work transnational youth perform online (Darvin, 2018; Lam and Wu, 2024) (Table 1).

**Table 1:** Key mediating mechanisms, structural conditions, and developmental outcomes in digital identity development among transnational youth.

Analytical dimension	Core elements in synthesized literature	Synthesized role in identity work	Educational/developmental implications
Mediating mechanisms: multilingual practices	Multilingual practices *(code-switching, mixed repertoires), translanguaging, hybrid language forms*	Appears to enable flexible positioning across audiences; tends to index belonging, legitimacy, and cultural affiliation	Tends to support language development, intercultural competence, and informal learning outside school; may also trigger linguistic hierarchies and exclusion
Narrative and discursive agency	*Counter-storytelling*, identity narratives, justice-oriented literacies, discursive reframing	Serves to challenge marginalizing representations; appears to articulate alternative selves and collective identities	Tends to enhance voice, agency, and psychosocial affirmation; may increase exposure to conflict, backlash, or harassment
Self-presentation strategies	*Strategic self-presentation*, selective visibility, boundary management, and differentiated platform use across audiences	Functions to manage context collapse and multiple audiences; appears to reduce perceived risk and support identity coherence across settings	Relates to impression management, wellbeing, and coping; may intensify self-monitoring and psychological stress under heavy surveillance
Digital spaces as third spaces	*Social media as third spaces*; dialogic interaction; transnational peer networks; culturally specific online spaces	Appears to provide arenas for identity experimentation and belonging beyond home/school/nation binaries	Tends to bridge formal and informal learning; appears to support peer-based identity exploration; may also reproduce online segregation
Structural enabling conditions	*Digital capital* (devices, connectivity, platform literacy), supportive social networks, institutional recognition of digital literacies	Tends to expand participation, visibility, and opportunities for identity exploration and self-presentation	Tends to increase access to learning resources and supportive communities; appears to strengthen belonging and self-efficacy
Structural constraining conditions	*Surveillance* (familial/institutional/state), platform governance, content moderation, *algorithmic visibility*, racialized/linguistic discrimination, geopolitical asymmetries (Noble, 2018; Eubanks, 2018; Gillespie, 2018)	Tends to produce self-censorship; shapes what is seen and valued; appears to limit agency and participation; tends to reinforce cultural hierarchies	May undermine wellbeing and identity integration; tends to reduce equitable learning opportunities; may reinforce existing social inequalities
Developmental outcomes: identity	*Hybrid identities*, negotiated cultural/national/religious/gender identities, multi-sited belonging	Appears to support identity integration across local/translocal/transnational contexts	Tends to promote adaptive identity development; may also intensify identity tension under strong structural constraints
Developmental outcomes: learning	Digital, linguistic, and cultural literacies; bridges between informal and formal learning contexts	Tends to convert participation into competencies and meaningful learning trajectories	Points toward the need for culturally responsive and digitally informed educational practices; highlights the importance of recognizing out-of-school literacies

**Figure 1:**
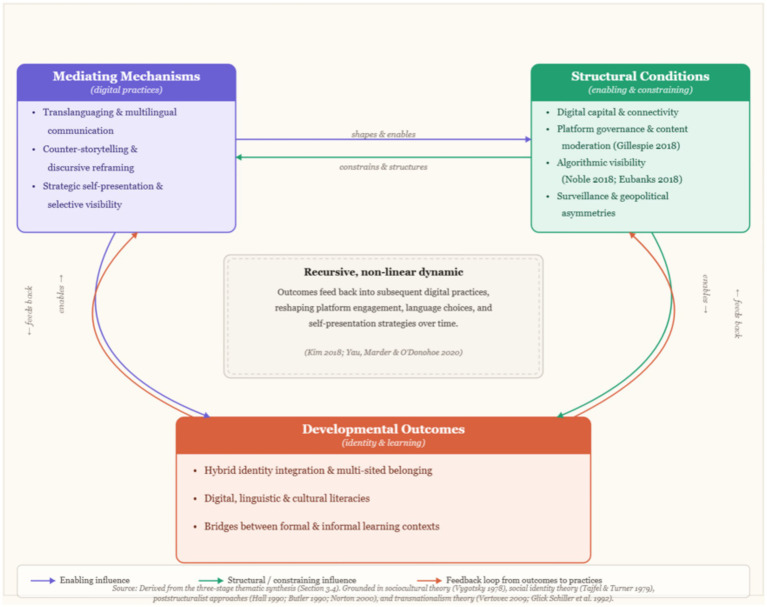
Integrative conceptual model of digital identity development among transnational youth.

Rows correspond to the five thematic clusters identified through the three-stage analytical procedure described in Section 3.4. Column 1 identifies the analytical dimension; Column 2 lists core elements identified across the synthesized literature; Column 3 describes the synthesized role of each dimension in identity work as it emerges across the reviewed studies; Column 4 specifies educational and developmental implications. Categories were derived inductively from the corpus and validated through constant comparison. Representative sources for each row are cited in the corresponding Results subsections (Sections 4.1–4.6). The table should be read in conjunction with Figure 1, which illustrates the recursive relationships among the three model components.

### 4.7 An integrative conceptual model of digital identity development among transnational youth

Building on the five thematic clusters I present above, I propose an integrative conceptual model that synthesizes the analytical findings into a coherent theoretical framework. The model is not a descriptive summary of prior studies, but a theory-building synthesis that explains how and under what conditions social media mediates identity development, learning, and belonging in transnational contexts.

I conceptualize digital identity development as a dynamic and recursive process emerging from three interrelated dimensions: mediating mechanisms, structural conditions, and developmental outcomes. Mediating mechanisms refer to the concrete digital practices through which transnational youth actively perform, negotiate, and reconfigure identities—translanguaging, counter-storytelling, and strategic self-presentation (Lam, 2009; Abokhodair, 2017; Kim, 2018). These practices unfold within structural conditions—access to digital capital, institutional recognition, surveillance, platform governance, algorithmic visibility, geopolitical asymmetries (Lam, 2014; Darvin, 2018; Noble, 2018; Sarnou, 2025)—that both enable and constrain identity work. The interaction between practices and conditions produces developmental outcomes: hybrid identity integration, multi-sited belonging, and bridges between informal and formal learning contexts (Mevsimler, 2021; Lam and Wu, 2024).

Crucially, these dimensions are not linearly ordered but recursively connected: outcomes feed back into subsequent digital practices, reshaping platform engagement, language choices, and self-presentation strategies over time. Experiences of recognition or marginalization may intensify counter-storytelling, alter language choices, or prompt shifts in platform use, thereby reconfiguring future identity trajectories (Kim, 2018; Yau et al., 2020). This recursive, non-linear logic—grounded in the sociocultural tradition’s emphasis on dynamic mediated learning (Vygotsky, 1978)—distinguishes my proposed model from prior frameworks that treat identity development as a predominantly linear process of acculturation or capital acquisition (cf. Lam, 2006; Darvin, 2018).

Figure 1 provides a schematic illustration of the integrative model. The figure depicts the three components as interconnected nodes with bidirectional arrows indicating recursive feedback loops. Labels are fully consistent with the terminology I use throughout Sections 3 and 4.

## 5 Discussion

In this section, I interpret the five thematic clusters and the integrative model in relation to existing scholarship on social media, transnational youth, and identity development. Rather than restating the themes, I focus on their theoretical significance, educational implications, and critical dimensions—connecting my synthesis findings to the theoretical traditions I establish in Section 2.0.

### 5.1 Social media as a formative context for identity development

The themes emerging from my synthesis reinforce and extend previous research by demonstrating that identity development among transnational youth is a dynamic and relational process shaped through participation in socially mediated environments—a finding consistent with sociocultural theory’s emphasis on mediated social action (Vygotsky, 1978; Wertsch, 1991). Social media emerges not merely as a supplementary communicative tool but as a formative context in which identity work unfolds (Lam, 2009; Kim, 2018; Yau et al., 2020). My synthesis advances the field by integrating educational perspectives more explicitly, showing that identity development through social media is inseparable from learning processes, language use, and participation in culturally meaningful practices (Darvin, 2018; Nantwi et al., 2017).

### 5.2 Reconsidering “third spaces” in digital and educational contexts

The conceptualization of social media as “third spaces” is strongly supported by the synthesized literature and is theoretically grounded in sociocultural accounts of learning and participation in communities of practice. These spaces appear to enable transnational youth to move beyond home–school–nation binaries and hybridize identity resources from multiple sociocultural contexts (Lam and Wu, 2024; Koyama, 2020). However, my synthesis cautions against romanticizing third spaces as inherently liberatory. They are also structured by platform affordances, algorithmic visibility regimes, and social hierarchies—features that reflect the political economy of digital platforms rather than the needs of their users (Leurs, 2008; Pang and Wang, 2020; Gillespie, 2018). Pedagogically, this implies that the value of digital spaces depends not on access alone but on how they are critically engaged within institutional settings (Eamer et al., 2014).

### 5.3 Language, multilingualism, and identity negotiation

The prominence of multilingual practices in my synthesis confirms that language remains a central semiotic resource for identity development in transnational digital environments—consistent with poststructuralist accounts of language as constitutive of, rather than merely expressive of, identity (Norton, 2000; Hall, 1990). Translanguaging and hybrid language use function as identity markers through which youth index belonging, authority, and legitimacy (Abdi, 2016; Lam, 2014). My findings support critiques of monolingual and deficit-oriented educational models and reinforce calls for frameworks that view multilingual digital practices as assets. Recognizing these practices can contribute to culturally responsive pedagogies that align with youths’ lived communicative realities (Lam, 2006; McKenzie, 2022).

### 5.4 Agency, constraint, and the dual nature of digital media

A key contribution of my synthesis lies in reconciling divergent findings regarding empowering versus constraining effects of social media. The synthesized literature indicates that digital media simultaneously enables agency and reproduces inequality—a duality shaped by structural conditions including access to digital resources, migration status, generational position, and societal reception of migrant groups (Darvin, 2018; Leurs and Ponzanesi, 2013). This duality cannot be understood without attending to the political economy of digital platforms.

Major commercial platforms operate under profit logics that prioritize engagement and data extraction over equitable participation. Content moderation systems apply culturally specific standards that disadvantage multilingual and minority-language expression (Noble, 2018; Eubanks, 2018). Recommendation algorithms amplify content that resonates with dominant audiences at the expense of minority voices (Gillespie, 2018). These features are constitutive of who can speak, who is heard, and what forms of self-representation are legible within these environments. Geopolitical asymmetries compound these dynamics: the dominance of US-origin platforms, uneven enforcement of content moderation across linguistic communities, and differential privacy protections across jurisdictions all shape how identity work can be conducted (Sarnou, 2025). My analysis aligns with critical perspectives that caution against universalizing claims about digital empowerment (Abokhodair, 2017; Sarnou, 2025) and underscores the need to situate identity development within broader relations of power and platform governance.

### 5.5 Educational implications and policy considerations

My synthesis indicates that social media can bridge formal and informal learning when institutions build upon the competencies youth develop online. When educational policies adopt narrow conceptions of digital literacy, however, they risk obscuring the complex identity work in digital environments (Nantwi et al., 2017; Lam and Wu, 2024). The following implications are offered at three differentiated levels.

#### 5.5.1 Implications for classroom teachers

Teachers can draw directly on the digital and multilingual practices I document in this synthesis. Integrating multilingual digital artifacts—student-produced social media content, translanguaged posts, or counter-narratives created in students’ home languages—into language arts, social studies, and intercultural education curricula constitutes a concrete application of culturally responsive pedagogy grounded in the sociocultural tradition (Vygotsky, 1978; Lam, 2014; McKenzie, 2022). Teachers should also be prepared to address platform governance, algorithmic visibility, and surveillance as components of critical digital literacy education, drawing on the structural analysis I develop in Section 4.5.

#### 5.5.2 Implications for institutional administrators

Schools and universities can formalize recognition of digital and multilingual literacy competencies acquired outside formal schooling by revising assessment frameworks, establishing institutional policies that recognize digital literacies as educationally consequential, and providing teachers with professional development resources focused on transnational students’ digital identity practices. At the higher education level, portfolio-based assessment mechanisms that document multilingual digital literacy competencies developed across formal and informal contexts would represent a meaningful institutional response (Darvin, 2018; Eamer et al., 2014).

#### 5.5.3 Implications for policymakers

Digital equity frameworks must address not only access to devices and connectivity, but also the structural conditions I document in this synthesis: surveillance, platform governance, and geopolitical asymmetries. Policymakers should consider regulatory frameworks that address algorithmic discrimination and unequal content moderation as educational equity concerns (Noble, 2018; Eubanks, 2018; Leurs, 2008). International policy coordination on platform governance standards—particularly with respect to multilingual content moderation and data privacy for youth—would represent a meaningful step toward reducing geopolitical asymmetries in digital participation (Sarnou, 2025).

### 5.6 Limitations and directions for future research

This study is not without limitations that I should clearly acknowledge. As a conceptual synthesis, my conclusions are shaped by the scope and distribution of existing studies. Several specific limitations warrant explicit acknowledgment:

- *Selection bias*: the corpus, while purposively constructed, cannot be exhaustive, and the possibility of overrepresentation of English-language and Western-context scholarship should be acknowledged. Non-English publications and scholarship from the Global South are underrepresented in the major databases I searched.
- *Disciplinary bias*: my synthesis draws primarily on scholarship from education, sociology, and applied linguistics, and may underrepresent perspectives from computer science, communication studies, or public health that could contribute additional dimensions to the analysis.
- *Absence of primary empirical validation*: I explicitly position the proposed integrative model as a theory-building contribution requiring empirical validation in future research. Its claims are grounded in patterns across the synthesized literature, not in direct empirical data collection.
- *Limitations of conceptual synthesis design*: my synthesis-based approach cannot fully account for the diversity of transnational youth experiences across regions, platforms, and migration contexts. The model should be understood as offering a generalizable analytical framework rather than a description of any particular population’s experience.

Future research would benefit from longitudinal and comparative designs examining identity development across migration stages and educational trajectories (Deroo and McClure, 2024; Xu, 2022). Further empirical work is needed on platform-specific dynamics, algorithmic visibility, and emerging technologies—including generative AI tools—in shaping identity formation among transnational youth. Empirical validation of the integrative model I propose in Section 4.7, and greater attention to underrepresented Global South contexts, represent priority directions (Chu, 2025; Mevsimler, 2021).

## 6 Conclusion

In this article, I examined the role of social media and digital media practices in shaping identity development among transnational youth from educational and cultural perspectives. Drawing on an analytical synthesis of interdisciplinary scholarship and grounded in four complementary theoretical traditions—sociocultural theory, social identity theory, poststructuralism, and transnationalism theory—I demonstrated that social media constitutes a formative environment in which transnational youth negotiate belonging, language, and identity across cultural, linguistic, and national boundaries.

My analysis identified key mechanisms including multilingual and translanguaging practices, hybrid identity construction, counter-storytelling, and strategic self-presentation. These practices enable youth to maintain simultaneous affiliations with local, translocal, and transnational communities, while navigating tensions related to cultural expectations, visibility, and social inequality. My synthesis underscored the dual and asymmetric nature of digital media: while offering opportunities for agency, voice, and learning, social media environments are structured by platform governance, algorithmic visibility (Noble, 2018; Eubanks, 2018; Gillespie, 2018), surveillance, geopolitical asymmetries, and unequal access to digital capital, all of which constrain developmental outcomes in unevenly distributed ways.

The central contribution of this study is the integrative triadic model I propose, which bridges educational and cultural perspectives and explicitly grounds the framework in the four theoretical traditions I outline in Section 2.0. By foregrounding the recursive, non-linear relationships among mediating mechanisms, structural conditions, and developmental outcomes, I advance a theoretically grounded and educationally applicable understanding of digital identity development. My model’s engagement with platform power, surveillance, and geopolitical asymmetry distinguishes it from prior frameworks and offers a critical corrective to technocentric accounts.

I acknowledge the study’s limitations—including selection bias, disciplinary bias, and the absence of primary empirical validation—and I position empirical validation of the proposed model as a priority for future research. Nonetheless, the integrative framework I develop offers an analytically coherent basis for guiding comparative empirical research, informing culturally responsive pedagogy, and supporting evidence-based policy in contexts of increasing digital mediation and transnational mobility.

## Data Availability

The original contributions presented in the study are included in the article/Supplementary material, further inquiries can be directed to the corresponding author.
