# Supplementary material for: Social media as a formative educational and cultural environment: an integrative conceptual framework for identity development among transnational youth
Source: Front Sociol. 2026 Jul 21;11:1834754. doi: 10.3389/fsoc.2026.1834754 (PMC13433208; doi:10.3389/fsoc.2026.1834754)
Supplement: Supplementary file 1 [file Table_1.docx]

# **Appendix B: Summary of Analytical Corpus**

**Table A2.** *Complete Summary of Analytical Corpus (42 Studies)*

*Note.* F = Foundational theoretical work; E = Empirical study; T = Theoretical/conceptual contribution; T/E = Combined theoretical and empirical contribution. Studies are ordered by thematic cluster contribution: foundational works first (nos. 1–6), followed by empirical and theoretical contributions organized by thematic relevance to the five analytical clusters identified in Section 3.4. All 42 studies in this table constitute the full analytical corpus retained following the three-stage screening procedure described in Sections 3.2–3.3 and illustrated in Figure A1. The table should be read in conjunction with Table 1 (Section 4.6) and Figure 1 (Section 4.7).

| **No.** | **Author(s) & Year** | **Type** | **Study Focus** | **Population / Context** | **Method** | **Key Contribution to Synthesis** |
| --- | --- | --- | --- | --- | --- | --- |
| **1** | Lam (2006) | **F** | Digital literacies and immigrant identity | Chinese/HK immigrant high school students, USA | Qualitative longitudinal | Foundational ecological framework for digital literacies and identity; introduced concept of new mediascapes as identity spaces |
| **2** | Lam (2009) | **E** | Multiliteracies on instant messaging | Chinese immigrant adolescent, USA | Qualitative case study | Demonstrated multilingual IM practices enabling negotiation of local, translocal, and transnational affiliations |
| **3** | Leurs (2008) | **F** | Digital diasporas and youth identity | Moroccan-Dutch youth (n=1408 survey, n=43 interviews) | Mixed methods | Foundational digital diasporas framework; documented generational and gender differences in digital identity practices |
| **4** | Leurs & Ponzanesi (2013) | **E** | Homeland and diaspora online | Moroccan-Dutch youth | Qualitative | Generational and gender transformations in diaspora digital practices; documented family brokering role |
| **5** | Darvin (2018) | **F** | Digital identity and educational inequality | Filipino migrant youth, Canada | Ethnographic/Mixed | Investment model of digital identity; critical analysis of class, social capital, and digital capital; foundational for structural constraints analysis |
| **6** | Yi (2009) | **E** | Adolescent literacy and identity construction | 1.5 generation transnational students | Qualitative | Examined how transnational students construct identity through digital and literacy practices across multiple communities |
| **7** | Wilding (2012) | **E** | Mediated culture in transnational spaces | Refugee youth, Australia (Melbourne) | Qualitative | Documented digital media mediating cultural negotiation for refugee youth; highlighted surveillance and access constraints |
| **8** | Kim (2018) | **E** | Migrant youth identity in new mediascape | Korean migrant adolescents | Qualitative ethnographic case study | Examined generational differences in digital identity formation; documented identity tensions between heritage and host contexts |
| **9** | Kim (2015) | **E** | Korean migrant youth identity in transnational social field | Korean migrant youth, Canada | Qualitative | Explored link between identity, transnationalism, and new media literacy among Korean diaspora youth |
| **10** | Lam & Wu (2024) | **E** | Chronotopes of transnational literacies | Chinese descent youth, urban high school, USA | Qualitative case study | Advanced chronotopic framework; documented how youth live and imagine social worlds in digital media practices |
| **11** | Abokhodair (2017) | **E** | Privacy and identity on Facebook | Saudi transnational youth | Mixed methods | Documented strategic privacy management and identity performance; analyzed surveillance effects on self-presentation |
| **12** | Sarnou (2025) | **T** | Digital colonization and youth | Algerian youth Gen Z/Alpha | Theoretical/Critical | Extended postcolonial framework to digital media; documented geopolitical asymmetries and algorithmic inequalities for Global South youth |
| **13** | Koyama (2020) | **T/E** | Transworlding in online schooling | Transnational youth in online school | Ethnographic | Developed concept of transworlding; showed how youth navigate multiple social worlds across digital and physical contexts |
| **14** | McKenzie (2022) | **E** | Digital media and cultural identity development | Hmong American emerging adults (M=21.94, n=17), USA | Mixed methods | Documented digital media as site for cultural identity; counter-storytelling supporting identity affirmation among minority youth |
| **15** | Yau, Marder & O'Donohoe (2020) | **E** | Social media and acculturation identity | International students (n=27, ages 18–25), UK | Qualitative interviews | Documented role of social media in acculturation; detailed strategic identity management and platform differentiation practices |
| **16** | Pang & Wang (2020) | **E** | Social media and acculturation process | International university students | Literature review/Theoretical | Examined social media dual role in promoting and constraining acculturation; documented platform-specific effects |
| **17** | Mevsimler (2021) | **E** | British Somali women online | Second-generation British Somali women | Qualitative | Documented translocal identity nexus; showed how digital media enables navigation of heritage and host contexts across generations |
| **18** | Nantwi, Chae & Natriello (2017) | **E** | Social media identities of African immigrants | African immigrant youth, USA | Qualitative | Documented social media identity practices; provided educational implications for multicultural educators |
| **19** | Deroo & Mohamud (2022) | **E** | Translanguaging and religious identity | Transnational immigrant Muslim youth | Qualitative longitudinal (2 years) | Examined critical conversations supporting transnational religious identity across time and space; documented translanguaging practices |
| **20** | Deroo & McClure (2024) | **E** | Transnational identity positioning | Students in United States and Ireland | Qualitative comparative case study | Comparative analysis of transnational identity positioning; documented contextual influences on identity negotiation |
| **21** | Xu (2022) | **E** | Social media and international student identity | Chinese international student | Qualitative case study | Explored impacts of social media on identity development; documented code-switching and strategic platform use |
| **22** | Solmaz (2020) | **E** | Transcultural practices as identity performance | International students in digital settings | Qualitative | Analyzed transcultural digital practices as identity performances; documented entextualization and translocalization processes |
| **23** | Eamer, Hughes & Morrison (2014) | **E** | Cultural border crossing through social networking | Bi-cultural students, Canada | Qualitative | Documented Ning-based telecollaboration facilitating cultural border crossing and identity exploration in educational settings |
| **24** | Ibold (2010) | **E** | Youth, internet use and cultural identity | Youth in Bishkek, Kyrgyzstan | Qualitative | Examined disjuncture between internet use and cultural identity formation in post-Soviet Central Asian context |
| **25** | Yoon (2016) | **E** | Mobile app platforms in transnational context | Young Koreans using KaTalk and Facebook | Qualitative | Analyzed media practice differences between domestic and transnational platforms; documented platform-specific identity affordances |
| **26** | McDaniel (2024) | **E** | Social media activism and justice-oriented literacies | Transnational youth activist, USA | Qualitative case study | Documented justice-oriented digital literacies; showed how social media activism constructs and affirms marginalized identities |
| **27** | Mallos (2020) | **E** | Greek migrant young adults' identity through new media | Greek migrant young adults | Participatory narrative inquiry | Combined public pedagogy, participatory narrative inquiry, and heteroglossia to examine digital identity construction |
| **28** | Prinsen, Haan & Leander (2015) | **E** | Networked identity among immigrant-background youth | Immigrant-background and native Dutch teens | Comparative qualitative | Conceptualized identity as a networked phenomenon; integrated social network analysis with sociological identity theories |
| **29** | Eide, Krøvel & Knudsen (2014) | **E** | Transnational orientations in global media landscape | Youth across multiple national contexts | Mixed methods | Examined transnational orientations and media use; documented how global media shapes youth identity and political engagement |
| **30** | Zhao (2023) | **E** | Social media in lives of young connected migrants | Young connected migrants | Qualitative | Documented how social media mediates belonging, identity, and connectivity for young mobile migrants in contemporary digital landscapes |
| **31** | Baan (2020) | **E** | Networked lives of immigrant-background teens | Immigrant-background and native Dutch teens | Comparative qualitative | Traced connections of learning across formal and informal digital contexts; documented differential access and network formation |
| **32** | Boumba (2014) | **E** | Media consumption of second-generation diaspora | Congolese diaspora youth, Brussels | Qualitative | Documented plural identity negotiation (double, African/Congolese, or cosmopolitan) through social network sites among diaspora youth |
| **33** | de Kool (2012) | **E** | Turkish youths and media preferences | Turkish-Dutch youth | Mixed methods | Examined Turkish-Dutch youth's media preferences; documented strong attachment to Turkish cultural media and digital identity expression |
| **34** | Abdi (2016) | **E** | Multidirectional language socialization | Canadian transnational child in Chinese school | Qualitative case study | Documented multidirectional language socialization; showed how transnational children navigate multiple linguistic worlds |
| **35** | Hinton & Putra (2020) | **E** | Indigenous language learning on social media | Indigenous youth (Lampung, Mayangna, Miskitu) | Qualitative | Examined digital activism for reclaiming indigenous languages; documented counter-hegemonic identity practices through social media |
| **36** | Willem (2010) | **E** | Digital media and ethno-cultural identity construction | Youth from diverse ethno-cultural backgrounds | Qualitative | Examined how youth use digital media to construct 'roots and routes'; documented hybrid identity construction processes |
| **37** | Cordova, Riva & Vittadini (2010) | **E** | Communication practices and immigrant adolescents | Immigrant adolescents | Mixed methods | Documented emerging communication practices; examined how digital media shapes developmental identity processes for immigrant youth |
| **38** | Dekker (2018) | **T/E** | Digital humanities and migration research | Migrant youth (methodological focus) | Methodological/Mixed | Examined methodological passages for studying digital identity; contributed to transparency and rigor in digital migration research |
| **39** | Lam (2014) | **E** | Literacy and capital in immigrant youths' online networks | Immigrant youth across multiple countries | Qualitative comparative | Documented relationship between literacy practices and capital accumulation in immigrant youth's transnational online networks |
| **40** | Chu (2025) | **E** | Cross-Strait identity formation among Taiwanese youth | Taiwanese youth | Survey/Mixed | Examined media exposure, political socialization, and civic engagement; documented digital media's role in cross-border identity formation |
| **41** | Gillespie (2018) | **F** | Platform governance and content moderation | N/A — theoretical/critical | Theoretical | Foundational analysis of custodians of the internet; documented hidden decisions in content moderation shaping digital participation |
| **42** | Noble (2018) & Eubanks (2018) | **F** | Algorithmic discrimination and digital inequality | N/A — theoretical/critical | Theoretical/Critical | Documented algorithmic oppression and automated inequality; grounded analysis of differential platform outcomes for racialized and minoritized users |

**Type codes:** F = Foundational work underpinning the theoretical framework (Section 2.0); E = Empirical study providing primary analytical evidence; T = Theoretical or conceptual contribution; T/E = Contribution combining theoretical and empirical elements. Full references for all 42 studies are provided in the References section.
