# Supplementary material for: Social media as a formative educational and cultural environment: an integrative conceptual framework for identity development among transnational youth
Source: Front Sociol. 2026 Jul 21;11:1834754. doi: 10.3389/fsoc.2026.1834754 (PMC13433208; doi:10.3389/fsoc.2026.1834754)
Supplement: Supplementary file 2 [file Table_2.docx]

**Table S1. *Search Strategy: Databases, Search Terms, and Corpus Construction Trajectory***

| **Database** | **Key Search Terms (in combination)** | **Date Range** | **Initial Results** |
| --- | --- | --- | --- |
| Google Scholar | ("social media" OR "digital media") AND ("identity development" OR "identity negotiation") AND ("transnational youth" OR "migrant youth" OR "immigrant youth" OR "refugee youth") | 2006–2025 | ~72 |
| Scopus | ("social media" OR "digital media") AND ("identity" OR "identity formation") AND ("transnational" OR "migrant" OR "immigrant") AND ("youth" OR "adolescent" OR "student") | 2006–2025 | ~48 |
| ERIC | ("digital media" OR "social media") AND ("identity" OR "multilingual") AND ("transnational" OR "migrant" OR "refugee") AND ("education" OR "learning") | 2006–2025 | ~38 |
| Web of Science | ("social media" OR "digital identity") AND ("transnational youth" OR "migrant youth" OR "international students") AND ("education" OR "cultural identity" OR "belonging") | 2006–2025 | ~22 |

*Note.* Additional search terms used in combination across databases included: translanguaging; hybrid identity; digital literacies; platform governance; algorithmic visibility; counter-storytelling; cultural negotiation; code-switching; digital capital; third spaces. Backward reference tracking of foundational studies (Lam 2006; Leurs 2008; Darvin 2018) yielded an additional ~18 sources reviewed at the full-text stage. Total initial pool: ~180 sources. Following title and abstract screening: 78 sources retained for full-text review. Following full-text assessment for analytical relevance, depth, and scholarly quality: 42 studies retained in the final analytical corpus. Conceptual saturation was operationalized as the point at which additional sources did not introduce new analytical categories or substantially alter the emerging thematic structure (Jabareen 2009; Torraco 2005).
